# Multimodal prototypical network for interpretable sentiment classification

**DOI:** 10.1038/s41598-025-19850-6

**Published:** 2025-10-26

**Authors:** Chenguang Song, Ke Chao, Bingjing Jia, Yiqing Shen

**Affiliations:** 1 Anhui Science and Technology University, Bengbu, 233000 China; 2 Johns Hopkins University, Baltimore, 21218 USA

**Keywords:** Multimodal sentiment analysis, Multimodal prototypical networks, Interpretability, Computational biology and bioinformatics, Engineering, Mathematics and computing

## Abstract

Recent advances in sentiment analysis have primarily focused on fusing multimodal information from video data, including visual, acoustic, and textual features, across temporal sequences. While great effort has been made to integrate or fuse information across modalities, less is known about the extent to which temporal segments contribute to model decisions. In addition, current interpretable methods, such as prototype networks, are primarily designed for uni-modal analysis and fail to handle the complex interactions between multiple modalities and temporal dependencies inherent in video data. To address the challenges, we propose **M**ulti**M**odal **P**rototypical **Net**works (MMPNet), which extends prototype-based interpretability to multimodal sentiment classification. Specifically, MMPNet can identify contributions of time-level features and leverage them to explain why a particular prediction was made, while also helping to find the relative importance of modality-level features. Experimental results show that MMPNet outperforms existing methods by 2.9% and 1.6% in accuracy on CMU-MOSI and CMU-MOSEI respectively, and achieves better interpretability.

## Introduction

Sentiment analysis, which focuses on identifying and interpreting human emotional attitudes towards various entities and situations, has traditionally centered on analyzing textual data^1–3^. However, the contemporary digital landscape has witnessed a change in how people express opinions, moving from purely textual formats to rich multimodal interactions that incorporate video, audio, and visual elements^4–6^. This evolution has led to multimodal sentiment analysis (MSA), a field that integrates multiple data streams to achieve a more complete understanding of human emotions and intentions^7–9^. MSA systems analyze textual content alongside audio signals and visual cues, which creates opportunities for more accurate and context-aware sentiment analysis^10–12^. Recent advances in MSA have produced various deep learning architectures that capture complex semantic relationships both within and across different modalities^13–16^, as well as innovative multimodal fusion strategies to generate effective representations from diverse data sources^17–19^.

Despite achieving promising classification accuracy, most existing MSA approaches rely on latent representations that lack interpretability. This opacity in decision making presents a limitation, as these models cannot explain why they assign specific sentiment classifications to particular inputs^20^. The interpretability of neural networks is equally important to their performance metrics, as it provides insights into the behavior of the model and the decision-making processes of MSA^21^. Understanding how these models arrive at their predictions is essential for ensuring reliability and improving generalization across diverse datasets. Consequently, some studies have begun to explore the contributions at the modality level to sentiment predictions, examining how different input channels influence the final classifications^22–26^. Although these investigations represent progress toward an explainable MSA, they have primarily focused on static feature attribution. The **gap** remains in understanding the temporal dynamics of the importance of the features and their relationship to the decision-making of the model across the time dimension.

Enhancing the interpretability of deep learning models has received increasing interest^27^. To make the decision-making process of the model more transparent and understandable, various strategies have been developed^28^. One particularly interesting approach is to construct an interpretable deep learning framework based on prototypes, such as the **Proto**typical **P**art **Net**work (ProtoPNet)^29^. In contrast to post-hoc methods, ProtoPNet and its variants are inherently interpretable methods that have been widely applied in interpretable image classification tasks^30^. In this work, we explore the possibility of extending the ProtoPNet from unimodal to multimodal tasks, specifically leveraging its self-interpretability advantages to construct an interpretable MSA model at the temporal feature level.

**Fig. 1:**
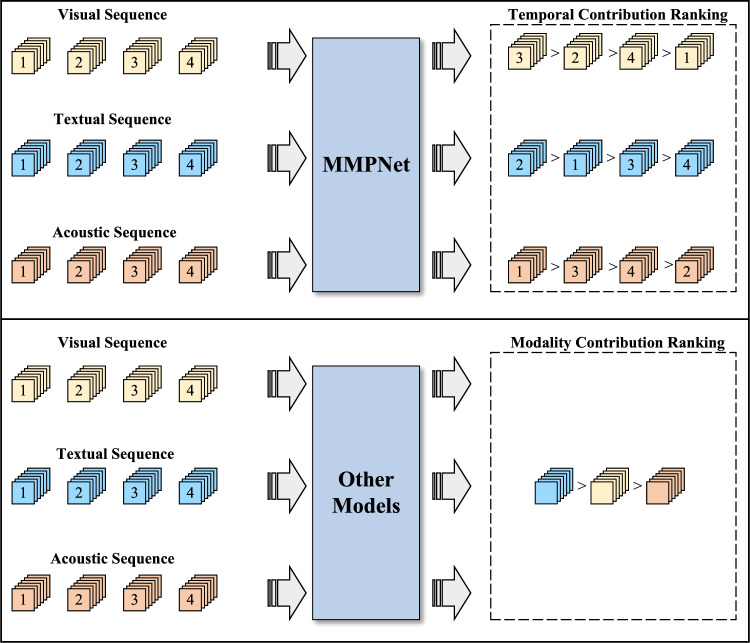
Comparison of contribution rankings between existing methods and MMPNet.

To bring this gap and inspired by ProtoPNet, we propose **M**ulti**M**odal **P**rototypical **Net**works (MMPNet), a prototype learning-based interpretable MSA method. MMPNet can identify contributions of time-level representations and leverage them to explain why a particular prediction was made, while also helping to find the relative importance of each modality. Due to the differences in representation space between images and temporal sequences, ProtoPNet cannot be directly applied to MSA tasks, nor to the temporal data. Innovatively, we convert multimodal temporal sequences into two-dimensional feature maps. Moreover, we design a local prototype branch for single modalities and an additional global prototype branch for multimodality, as the local branch alone may fail to capture modality interactions and be insufficient for a comprehensive analysis of sentimental cues. Figure 1 illustrates the key difference in feature contribution quantization between traditional multimodal models (bottom) and our proposed MMPNet (Top). For MMPNet, temporal contribution rankings are provided for each modality, including visual sequence (e.g., “$3> 2> 4 > 1$”), textual sequence (e.g., “$2> 1> 3 > 4$”), and acoustic sequence (e.g., “$1> 3> 4 > 2$”), enabling fine-grained quantification of time-level feature contributions . In contrast, existing methods only present modality-level contribution rankings (e.g., “ textual sequence > visual sequence > acoustic sequence”) without temporal granularity. Therefore, the **main differences** between MMPNet and existing research can be summarized as follows.

- MMPNet models and interprets the contributions of temporal-multimodal features to sentiment classification at both temporal and modality levels, while prior studies have focused solely on modality-level interpretability.
- MMPNet is specifically designed to process multimodal temporal data, while ProtoPNet and its variants are mainly used to process image data.

The major contributions are three-fold.

- We explore a novel problem, namely how multimodal temporal features contribute to multimodal sentiment classification. By investigating the temporal dynamics of feature importance and their relationship to model decisions, we address a gap in understanding the interpretability of MSA models.
- We introduce MMPNet, a self-interpretable deep learning framework, to the domain of multimodal sentiment classification. MMPNet skillfully adapts ProtoPNet to handle the complexities of multimodal temporal data, enabling the identification of modality-specific and cross-modal contributions to sentiment classification. In addition, MMPNet jointly learns the modality-specific information and multimodal interaction information through the local prototype branch and the global prototype branch, respectively.
- We conduct extensive experiments on publicly available datasets to demonstrate the effectiveness of MMPNet. Our experimental results show MMPNet’s ability to achieve state-of-the-art performance while providing transparent explanations for its predictions at the temporal feature level.

## Related works

### Interpretable deep learning

The field of deep neural network (DNN) interpretability focuses on making model decision-making processes transparent and comprehensible^31^. As DNNs become increasingly complex, the need to understand their internal mechanisms has sparked extensive research into interpretability methods^32^. Interpretability approaches in deep learning can be categorized into two main branches based on when explanations are generated: (1) intrinsic interpretability and (2) post-hoc interpretability.

Intrinsic interpretability methods incorporate explanatory mechanisms directly into the model architecture^33^. Specifically, these self-explainable models include decision trees^34^, prototypical networks like ProtoPNet^29^, and concept-based learning approaches^35^. The explanations emerge naturally from the model’s structure and training process, making them integral to the model’s operation.

In contrast, post-hoc interpretability methods analyze trained models by introducing external explanation frameworks^36^. Notable examples include Local Interpretable Model-agnostic Explanations (LIME)^37^ and SHapley Additive exPlanations (SHAP)^38^. These approaches typically employ feature perturbation techniques or analyze feature contributions to understand model predictions^33^. While effective, post-hoc methods may not capture the full complexity of the model’s decision-making process since they examine the model after training.

Our proposed MMPNet extends the architecture of ProtoPNet to address the specific challenges of MSA. By inheriting and adapting ProtoPNet’s self-interpretable design principles, MMPNet provides built-in explanations for its predictions through learned prototypes.

### Interpretable multimodal sentiment analysis

The field of multimodal sentiment analysis (MSA) has evolved substantially through the integration of various fusion methods that can effectively combine different modalities^39–41^. While these approaches have achieved improved performance metrics in sentiment classification tasks, their reliance on complex neural architectures has resulted in poor interpretability. Consequently, making MSA models more transparent and interpretable has garnered increasing attention in recent years^42,43^.

An early attempt came from the multimodal factorized model (MFM)^44^, which pioneered the analysis of modality interactions during the training process. However, MFM’s capabilities were primarily confined to analyzing trimodal interaction features, lacking insights into how individual modalities and their dynamic interactions contribute to sentiment predictions. Subsequent research advanced with the introduction of multimodal routing (MURO) by Tsai et al.^22^. MURO integrated principles from capsule networks^45^ with concept-based explainable AI approaches^46^. By leveraging concept learning, where concepts represent high-level abstractions of prediction labels^47^, MURO implemented a dynamic routing mechanism to determine the relationship between multimodal features and classification outcomes. The learned routing coefficients provided insights into feature importance, establishing a framework for MSA model interpretation. Building upon MURO’s foundation, Wu et al.^23^ developed the interpretable multimodal capsule fusion (IMCF) model, which enhanced interpretability through a hierarchical architecture.

Despite these advances in modality-level interpretability, a fundamental gap remains in understanding temporal dynamics. Specifically, how temporal features across different modalities influence sentiment predictions over time remains unexplored. Our proposed MMPNet addresses this limitation by introducing a novel framework that explicitly models and interprets the contributions of temporal-multimodal features to sentiment classification.

## Problem formulation

This section formalizes the mathematical framework for interpretable multimodal sentiment classification. Our approach transforms traditional sentiment analysis from a **regression task** to a **classification problem**, allowing for more discrete and interpretable sentiment categories while maintaining the rich information from multiple modalities. We consider three primary modalities in our analysis, namely the visual, textual, and acoustic modalities. Let $\mathscr {X} = {\mathscr {X}_v ,\mathscr {X}_t ,\mathscr {X}_a}$ represent the multimodal input feature sequences, where subscripts *v*, *t*, and *a* denote visual, textual, and acoustic modalities, respectively. Each modality’s temporal sequence is represented as:

1 $$\begin{aligned} {\left\{ \begin{array}{ll} \mathscr {X}_v & = \{ \textbf{x}_v^{1}, \cdots , \textbf{x}_v^{i}, \cdots ,\textbf{x}_v^{L_v} \} \in {\mathbb {R}^{{L_v} \times {d_v}}}\\ \mathscr {X}_t & = \{ \textbf{x}_t^{1}, \cdots , \textbf{x}_t^{i}, \cdots ,\textbf{x}_t^{L_t} \} \in {\mathbb {R}^{{L_t} \times {d_t}}}\\ \mathscr {X}_a & = \{ \textbf{x}_a^{1}, \cdots , \textbf{x}_a^{i}, \cdots ,\textbf{x}_a^{L_a} \} \in {\mathbb {R}^{{L_a} \times {d_a}}} \end{array}\right. } \end{aligned}$$

where ${L_{\left( m\right) }}$ represents the sequence length and ${d_{\left( m\right) }}$ denotes the feature representation dimension for each modality $m \in \{ v,t,a\}$. Note that $d = {d_{\left( m\right) }} = d_v = d_a = d_t$ in this paper. For each temporal feature sequence $\mathscr {X}_m$, our objective is to learn a rank list $\mathscr {R}{(m)}$ that orders temporal features $\textbf{x}_{m}^{i}$ based on their contribution to the final sentiment prediction $\hat{y}$.

Now, we can define the problem of “**interpretable multimodal sentiment classification**”. Given a multimodal signal sequence $\mathscr {X}$ and a prototype layer $\mathscr {G}_{\textbf{p}}$, we aim to learn a multimodal sentiment classification function: ${\mathscr {F}}:\mathscr {F}\left( {\mathscr {X},{\mathscr {G}_\textbf{p}}} \right) \rightarrow \left( {\hat{y},{\mathscr {R}{\left( m \right) }}} \right)$. This function should maximize prediction accuracy while ensuring that the most interpretable and influential temporal segments are ranked highest in $\mathscr {R}_{(m)}$. This formulation aligns with our goal of providing transparent reasoning about how different modalities and temporal segments contribute to sentiment classification, as outlined in our prototype-based approach.

## Methods

### Overall framework

**Fig. 2:**
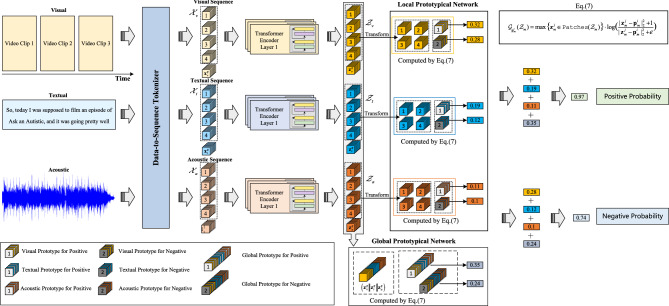
Overview of the MMPNet architecture for interpretable multimodal sentiment analysis.

As illustrated in Fig. 2, MMPNet processes multimodal input data through four main components: (1) a data-to-sequence tokenizer that transforms raw visual, textual, and acoustic inputs into standardized feature sequences; (2) modality-specific transformer encoders that generate both local sequential features and global token embeddings; (3) a dual-branch prototype network comprising local prototypical networks (LPN) for modeling modality-specific temporal patterns and a global prototypical network (GPN) for capturing cross-modal interactions; and (4) a sentiment predictor that combines similarity scores from both branches to generate interpretable sentiment classifications. The data-to-sequence tokenizer first transforms raw video, text, and audio inputs into their respective token embedding sequences (${\mathscr {X}_v}$, ${\mathscr {X}_t}$, ${\mathscr {X}_a}$). These sequences are then processed through dedicated transformer encoder networks for each modality.

During encoding, the transformers generates two types of representations: (1) local sequential features ($\mathscr {Z}_{v}$, $\mathscr {Z}_{t}$, $\mathscr {Z}_{a}$) and (2) global token embeddings ($\textbf{x}^{g}_{v}$, $\textbf{x}^{g}_{t}$, $\textbf{x}^{g}_{a}$). The local sequential representations ($\mathscr {Z}_{v} \in \mathbb {R}^{L_v \times {d_v}}$, $\mathscr {Z}_{t} \in \mathbb {R}^{L_t \times {d_t}}$, $\mathscr {Z}_{a} \in \mathbb {R}^{L_a \times {d_a}}$) preserve temporal information for each modality, where ${d_{(m)}}$ is the hidden dimension of the transformer encoder. The global token embeddings ($\textbf{x}^{g}_{v}, \textbf{x}^{g}_{t}, \textbf{x}^{g}_{a} \in \mathbb {R}^{d_{(m)}}$) aggregate information across the entire sequence of each modality through attention mechanisms, providing a comprehensive modality-specific representation. Specifically, MMPNet employs a dual-branch prototype architecture. The local branch processes each modality’s sequential representations independently through modality-specific prototype networks. These networks compute similarity scores between the input features and learned prototype embeddings ($\mathscr {P}_{v} \in \mathbb {R}^{K_v \times {d_v}}$, $\mathscr {P}_{t} \in \mathbb {R}^{K_t \times {d_t}}$, $\mathscr {P}_{a} \in \mathbb {R}^{K_a \times {d_a}}$), where $K_m$ denotes the number of prototypes for modality $m\in \{v,t,a\}$. This process reveals the interpretability of temporal-modal patterns by identifying which prototype patterns are most similar to the input sequences. Concurrently, the global branch concatenates the modality-specific global embeddings ($\texttt {Concat}[\textbf{x}^{g}_{v}; \textbf{x}^{g}_{t}; \textbf{x}^{g}_{a}] \in \mathbb {R}^{3d}$) to capture cross-modal interactions through a global prototype network. This branch enables the model to learn higher-level multimodal patterns that might not be visible when examining each modality in isolation.

The final sentiment prediction is generated by combining the similarity scores from both local and global prototype branches through fully-connected layers. This dual-branch architecture enables MMPNet to provide interpretable insights at both the modality-specific temporal level and the integrated multimodal level, addressing the challenges outlined in^20^ and^48^ regarding the need for explainable multimodal sentiment analysis.

### Data-to-sequence tokenizer

The data-to-sequence tokenizer module transforms raw multimodal inputs into standardized representations suitable for downstream processing. Following established approaches in multimodal sentiment analysis^22,23^, we employ specialized feature extractors for each modality: Facet for visual features, GloVe word embeddings for textual features, and COVAREP for acoustic features. These extractors generate initial feature vectors $\texttt {RawData}_m \in \mathbb {R}^{L_m \times d_m^{\text {raw}}}$ for each modality $m \in \{v,t,a\}$, where $d_m^{\text {raw}}$ represents the original feature dimension specific to each modality’s extractor. To ensure consistent representation across modalities while preserving temporal information, we process these initial features through modality-specific 1D temporal convolutional networks. Each $\texttt {Conv1D}_m$ consists of a one-dimensional convolutional layer with kernel size *k*, followed by batch normalization and ReLU activation. This transformation can be formally expressed as:

2 $$\begin{aligned} {\mathscr {X}_m} = \texttt {Conv1D}_m(\texttt {RawData}_m), \quad m \in \{v, t, a\}, \end{aligned}$$

where ${\mathscr {X}_m} \in \mathbb {R}^{L_m \times d}$ represents the tokenized sequence for modality *m*. Each sequence maintains its original temporal length $L_m$ while being projected into a shared embedding space of dimension *d*. This standardization ensures that all modalities can be effectively processed by subsequent transformer encoders while retaining their temporal characteristics.

### Feature encoding

The multimodal feature sequences ${\mathscr {X}}_m$ obtained from the data-to-sequence tokenizer contain rich temporal information that needs to be effectively captured and contextualized. To accomplish this, we employ separate transformer encoder networks for each modality to model long-range dependencies within the sequences. For each modality $m \in \{v,t,a\}$, we first augment the input sequence with a learnable global token embedding $\textbf{x}^g_m \in \mathbb {R}^d$. This global token serves as a modality-specific aggregator that can attend to and summarize information from the entire sequence. The augmented input sequence is created by prepending the global token to the original sequence:

3 $$\begin{aligned} \widetilde{\mathscr {X}}_m = \texttt {Concat}[\textbf{x}^g_m; \mathscr {X}_m] \in \mathbb {R}^{(L_m + 1) \times d}. \end{aligned}$$

The augmented sequence $\widetilde{\mathscr {X}}_m$ is then processed through a transformer encoder with $\eta _m$ layers, which can be formally expressed as:

4 $$\begin{aligned} {[}\textbf{z}^g_m; \mathscr {Z}_m] = \text {Transformer}_m(\widetilde{\mathscr {X}}_m), \quad m \in \{v,t,a\} \end{aligned}$$

where $\mathscr {Z}_m = \{\textbf{z}^1_m, \dots , \textbf{z}^{L_m}_m\} \in \mathbb {R}^{L_m \times d}$ represents the encoded sequence features, and $\textbf{z}^g_m \in \mathbb {R}^d$ is the updated global token representation. Each transformer layer employs multi-head self-attention mechanisms followed by feed-forward networks to capture complex temporal dependencies. The encoded representations serve two complementary purposes in our framework. The sequence features $\mathscr {Z}_m$ preserve detailed temporal information that will be processed by the local prototype branch to capture fine-grained patterns within each modality. Meanwhile, the global tokens $\textbf{z}^g_m$ provide condensed modality-specific representations that will be used by the global prototype branch to model high-level cross-modal interactions.

### Multimodal prototypical networks

The core of our framework is the Multimodal Prototypical Network (MMPNet), which consists of local and global prototype branches designed to capture both modality-specific temporal patterns and cross-modal interactions. Each branch plays a distinct role in achieving interpretable sentiment analysis. The local prototype network (LPN) processes the encoded sequences $\mathscr {Z}_m \in \mathbb {R}^{L_m \times d}$ from each modality $m \in \{v,t,a\}$ independently. For each modality, we learn a set of prototypes $\mathscr {P}_m = [\textbf{p}^1_m, \dots , \textbf{p}^{K_m}_m] \in \mathbb {R}^{{K_m} \times d}$, where $K_m$ is the number of prototypes per modality and each prototype $\textbf{p}^i_m \in \mathbb {R}^d$ represents a learned temporal pattern specific to that modality *m*. The LPN computes similarity scores between these prototypes and the encoded sequences:

5 $$\begin{aligned} \textbf{s}_m = \texttt {LPN}(\mathscr {Z}_m, \mathscr {P}_m) \in \mathbb {R}^{K_m}, \quad m \in \{v,t,a\} \end{aligned}$$

where $\textbf{s}_m = [s^1_m, \dots , s^{K_m}_m]$ contains the similarity scores for each prototype. For sake of simplicity, we set $K = K_v = K_t = K_a$. The global prototype network (GPN) processes the concatenated global token representations $[\textbf{z}^g_v; \textbf{z}^g_t; \textbf{z}^g_a] \in \mathbb {R}^{3d}$ to capture cross-modal patterns. We first apply a learnable transformation with parameters $\textbf{W}_m \in \mathbb {R}^{3d \times 3d}$ and $\textbf{b}_m \in \mathbb {R}^{3d}$, followed by ReLU activation $\sigma (\cdot )$. The transformed representation is then compared against global prototypes $\mathscr {P}_m^g \in \mathbb {R}^{K \times 3d}$:

6 $$\begin{aligned} \textbf{s}_m^g = \texttt {GPN}\Big ( \sigma ([\textbf{z}^g_v; \textbf{z}^g_t; \textbf{z}^g_a]\textbf{W}_m + \textbf{b}_m), \mathscr {P}_m^g\Big ) \in \mathbb {R}^K. \end{aligned}$$

**Fig. 3:**
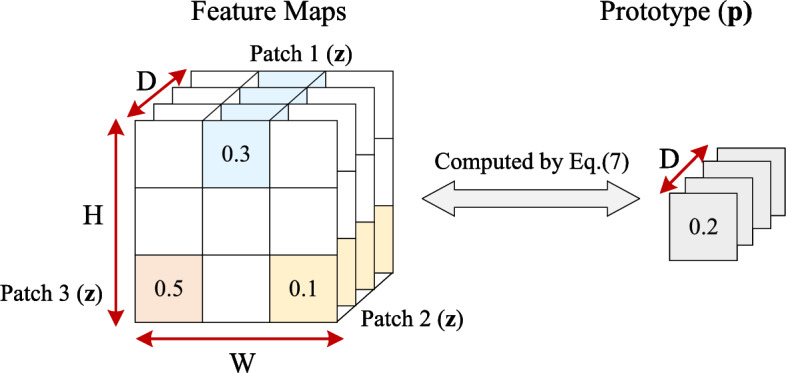
Illustration of the prototype similarity computation mechanism in MMPNet.

To adapt the prototype learning mechanism from spatial to temporal domain, we transform the temporal sequences into 2D feature maps. This transformation is important for applying prototype-based learning to temporal data. As shown in Fig. 3, the feature maps are divided into overlapping temporal patches, where each patch represents a segment of the temporal sequence. The similarity between a prototype and an input feature map is calculated to measure the distance between the prototype and the most similar patch within the feature map. The similarity score incorporates both the maximum similarity across all patches and a logarithmic scaling factor to ensure stable gradient flow during training. For each modality, we reshape $\mathscr {Z}_m \in \mathbb {R}^{L_m \times d}$ into $\mathbb {R}^{\lambda \times (L_m-\lambda ) \times d}$, where $\lambda$ is a hyperparameter controlling the temporal receptive field. This reshaping creates overlapping temporal segments that can be compared with prototypes. For computing similarity scores, we use a modified version of the traditional prototype network approach. As shown in Fig. 3, for each prototype $\textbf{p}^i_m$ and temporal sequence $\mathscr {Z}_m$, we compute:

7 $$\begin{aligned} \mathscr {G}_{\textbf{p}^i_m}(\mathscr {Z}_m) = \max \Big \{\textbf{z}^j_m \in \texttt {Patches}(\mathscr {Z}_m)\Big \} \cdot \log \Big (\frac{|\textbf{z}^j_m - \textbf{p}^i_m|^2_2 + 1}{|\textbf{z}^j_m - \textbf{p}^i_m|^2_2 + \varepsilon }\Big ) \end{aligned}$$

where $s^i_m = \mathscr {G}_{\textbf{p}^i_m}(\mathscr {Z}_m)$ represents the similarity score between prototype $\textbf{p}^i_m$ and the most similar temporal segment in sequence $\mathscr {Z}_m$. The $\texttt {Patches}(\mathscr {Z}_m)$ function extracts all possible temporal segments of length $\lambda$ from the sequence, and $\varepsilon$ is a small constant for numerical stability. This similarity computation identifies the temporal segments that best match each prototype pattern.

### Sentiment prediction

Building upon the multimodal prototype representations, we formulate sentiment analysis as a classification task that maps emotional expressions to discrete sentiment categories, rather than predicting continuous sentiment values through regression. The final sentiment prediction combines the similarity scores from both local and global prototype branches. We aggregate the similarity vectors from each modality ($\textbf{s}_v$, $\textbf{s}_t$, $\textbf{s}_a$) and the global branch ($\textbf{s}_m^g$) through element-wise addition, followed by a fully connected layer with weight matrix $\textbf{W} \in \mathbb {R}^{K \times \mathscr {C}}$, where $\mathscr {C}$ denotes the number of sentiment classes:

8 $$\begin{aligned} \hat{\textbf{y}} = \text {softmax}(\sum _{m\in \{v,t,a\}}(\textbf{s}_m + \textbf{s}_m^g)\textbf{W}). \end{aligned}$$

The output $\hat{\textbf{y}} = [\hat{y}_1, \dots , \hat{y}_c, \dots ,\hat{y}_{\mathscr {C}}]$ represents the predicted probability distribution over sentiment classes, where $\hat{y}_c$ indicates the probability of class *c*. To ensure interpretability, we establish a direct connection between prototypes and sentiment classes. For each class *c*, we allocate a subset of prototypes $\mathscr {P}^c_m \subseteq \mathscr {P}_m$, where the number of prototypes per class is chosen to be divisible by the total number of prototypes *K*. The weight matrix $\textbf{W}$ is structured to reflect this prototype-class association. For each class *c* and weight $w^{(i,c)} \in \textbf{W}$, we set:

9 $$\begin{aligned} w^{(i,c)} ={\left\{ \begin{array}{ll} 1 & \text {if } \textbf{p}^i_{(\cdot )} \in \mathscr {P}^c_{(\cdot )} \\ -\frac{1}{\mathscr {C}} & \text {if } \textbf{p}^i_{(\cdot )} \notin \mathscr {P}^c_{(\cdot )} \end{array}\right. }. \end{aligned}$$

The model is trained using cross-entropy loss between the predicted distribution $\hat{\textbf{y}}$ and the true distribution $\textbf{y}$:

10 $$\begin{aligned} \mathscr {L}(\Theta ) = -\sum _{c=1}^{\mathscr {N}} y_c \log (\hat{y}_c) \end{aligned}$$

where $\Theta$ represents all learnable parameters in MMPNet. This formulation ensures that each sentiment class is characterized by a specific set of prototypes, enabling interpretable predictions by revealing which temporal patterns and cross-modal interactions contribute to each sentiment classification.

## Experiments

### Datasets and evaluation metrics

We evaluate MMPNet on two widely-adopted multimodal sentiment analysis benchmarks: CMU-MOSI^49^ and CMU-MOSEI^50^. Both datasets contain aligned multimodal sequences spanning text, visual, and audio modalities extracted from YouTube movie reviews.

- CMU-MOSI: The CMU-MOSI dataset^49^ comprises 2,199 video clips sourced from 93 distinct YouTube movie reviews. Each clip is annotated with a continuous sentiment intensity score ranging from $-3$ (strongly negative) to $+3$ (strongly positive). For our binary classification task, we categorize samples with scores $\ge 0$ as positive sentiment and those with scores $<0$ as negative sentiment.
- CMU-MOSEI: Building upon CMU-MOSI, the CMU-MOSEI dataset^50^ offers a substantially larger collection of 23,453 video segments extracted from 5,000 videos. Similar to CMU-MOSI, each segment carries a sentiment intensity score between $-3$ and $+3$. We apply the same binary classification scheme, with samples scored $\ge 0$ classified as positive sentiment and those scored $<0$ as negative sentiment.

For both datasets, we utilize the aligned multimodal sequences provided in the preprocessed data and follow the train/validation/test splits established in previous work^51^. Table 1 presents the detailed statistics of both benchmarks. Given our focus on binary sentiment classification rather than regression, we evaluate model performance using four standard metrics: binary classification accuracy (positive/negative sentiments), precision, recall, and $F_1$ score.

**Table 1:** Data split distribution across training, validation, and test sets for the CMU-MOSI and CMU-MOSEI multimodal sentiment analysis benchmarks.

**Dataset**	**Train**	**Valid**	**Test**	**Total**
CMU-MOSI	1,284	229	686	2,199
CMU-MOSEI	16,326	1,871	4,659	22,856

### Experimental setup

We implement all experiments in PyTorch and executed on an NVIDIA GeForce RTX 3090 Ti GPU. For optimization, we employ the Adam optimizer with an initial learning rate of $5e^{-4}$. The batch size is configured differently for each dataset to accommodate their varying sizes: 256 for CMU-MOSEI and 40 for CMU-MOSI. The architecture of MMPNet incorporates the following hyperparameters. The transformer encoder network consists of 5 layers with 5 attention heads in each self-attention mechanism. For the prototype learning component, we set the number of prototypes *K* to 40, which provides sufficient capacity to capture diverse multimodal patterns while maintaining computational efficiency.

### Compared methods

Our proposed MMPNet leverages multimodal information from textual, visual, and acoustic sources to perform sentiment classification. To evaluate its effectiveness, we conduct comprehensive comparisons against both single-modality and multimodal approaches.

**Single modality models.** For each modality, we implement a transformer-based architecture as the single-modality baselines. The processing pipeline consists of three key steps. First, the data-to-sequence tokenizer generates the initial representations ($\mathscr {X}_m$) for each modality $m \in \{t,v,a\}$. These representations are then processed through modality-specific multi-layer transformer encoder networks^52^. Finally, the global token embedding ($\textbf{x}^g_m$) from each transformer encoder is passed through a fully-connected layer with softmax activation to generate the sentiment predictions. This consistent architecture across modalities enables fair comparison while highlighting the unique contributions of each information stream. The textual model captures semantic patterns in language, the visual model processes facial expressions and gestures, and the acoustic model analyzes speech characteristics such as tone and prosody. By establishing these single-modality performance benchmarks, we can better understand how MMPNet’s multimodal fusion and prototype learning mechanisms improve upon unimodal approaches.

**Multimodal models.** We also compare MMPNet against several multimodal architectures that represent different approaches to feature fusion and cross-modal interaction modeling. These methods can be broadly categorized into early fusion, late fusion, and advanced fusion approaches. Early fusion methods combine features at the input level before sequence processing. The Early Fusion LSTM (EF-LSTM) model concatenates the multimodal feature sequences ($\mathscr {X}_v$, $\mathscr {X}_t$, $\mathscr {X}_a$) before processing through an LSTM network^53^, followed by a fully-connected layer with softmax activation. Similarly, Early Fusion Transformer (EF-Transformer) applies the same concatenation strategy but utilizes a transformer encoder^52^ for sequence modeling. Late fusion approaches process each modality independently before combining their representations. Late Fusion LSTM (LF-LSTM) employs separate LSTM networks^53^ for each modality, while Late Fusion Transformer (LF-Transformer) uses independent transformer encoders^52^. In both cases, the modality-specific representations are concatenated before final classification. Other fusion strategies are implemented in several recent architectures. The Recurrent Attended Variation Embedding Network (RAVEN)^54^ dynamically modulates word representations using visual and acoustic context. The Multimodal Factorization Model (MFN)^55^ employs delta-attention and multi-view gated memory to model intricate cross-modal interactions. The Multimodal Transformer (MULT)^51^ leverages cross-modal transformer networks to iteratively enhance representations using complementary information from other modalities.

**Interpretable multimodal models.** Finally, we also compare against two interpretable multimodal models. Multimodal Routing (MURO)^22^ employs dynamic routing to quantify modality-specific contributions to the final prediction. The Interpretable Multimodal Capsule Fusion (IMCF)^23^ extends this approach with a hierarchical routing mechanism. While these models provide modality-level interpretability, our MMPNet advances the state-of-the-art by offering interpretability at both the temporal and multimodal levels through prototype learning.

For fair comparison, we adapt all regression-based models (RAVEN, MULT, MURO, and IMCF) to the classification setting by replacing their final prediction layers with softmax classification layers and using cross-entropy loss, while maintaining their core architectural components.

### Comparison on model performance

Our experimental evaluation demonstrates the effectiveness of MMPNet compared to state-of-the-art multimodal sentiment classification methods. Table 2 presents a comprehensive evaluation of MMPNet against other methods on the CMU-MOSI dataset, revealing several findings regarding model performance and interpretability. The experimental results demonstrate MMPNet’s superior performance, achieving the highest overall accuracy of 75.1%, surpassing both traditional and interpretable baseline methods. This represents a notable improvement of 2.9% points over MULT (72.2%), the second-best performing model. The performance gain is particularly meaningful as MMPNet maintains full interpretability while outperforming non-interpretable approaches. A detailed analysis of sentiment-specific metrics reveals MMPNet’s balanced performance across both positive and negative sentiment classifications. For positive sentiment detection, MMPNet achieves the highest precision (71.9%) and F1-score (72.3%), though EF-Transformer shows slightly better recall (77.2%). In negative sentiment detection, MMPNet demonstrates robust performance with the highest recall (77.0%) and F1-score (77.4%), while maintaining competitive precision (77.7%, second only to EF-Transformer’s 77.8%). Comparing against other interpretable methods, MMPNet also outperforms both MURO and IMCF across most metrics. While MURO and IMCF achieve respectable F1-scores for negative sentiment (73.6% and 74.1% respectively), MMPNet’s enhanced ability to capture temporal-multimodal patterns through prototype learning leads to more accurate predictions overall. The single-modality baselines’ performance underscores the importance of multimodal fusion, with the textual modality achieving the highest unimodal accuracy (70.7%). However, MMPNet’s superior performance (75.1%) demonstrates that effective integration of multiple modalities, combined with temporal-multimodal prototype learning, can substantially improve sentiment classification accuracy while maintaining interpretability (Table 2).

**Table 2:** Performance comparison on the CMU-MOSI dataset. Results show accuracy (‘Acc.’), precision (‘Prec.’), recall (‘Rec.’), and F$_{1}$ scores for both positive and negative sentiment classifications. Methods marked with $^\dag$ are inherently interpretable, providing transparency in their decision-making process through built-in mechanisms. Bold and underlined values indicate the best and second-best performances respectively.

Method	Acc.	Positive			Negative		
		Prec.	Rec.	F$_{1}$	Prec.	Rec.	F$_{1}$
Textual	0.707	0.659	0.717	0.686	0.753	0.699	0.725
Visual	0.525	0.493	0.769	0.601	0.657	0.359	0.464
Acoustic	0.507	0.463	0.629	0.533	0.576	0.409	0.478
EF-LSTM	0.667	0.605	0.733	0.663	0.739	0.612	0.670
LF-LSTM	0.718	0.678	0.700	0.689	0.751	0.731	0.741
EF-Transformer	0.703	0.639	**0.772**	0.699	**0.778**	0.646	0.706
LF-Transformer	0.719	0.665	0.749	0.704	0.774	0.694	0.732
RAVEN	0.672	0.659	0.693	0.676	0.686	0.652	0.669
MFN	0.676	0.585	0.657	0.619	0.747	0.685	0.714
MULT	0.722	0.668	0.752	0.708	0.776	0.697	0.734
MURO$^{\dag }$	0.703	0.676	0.645	0.660	0.723	0.749	0.736
IMCF$^{\dag }$	0.713	0.681	0.674	0.678	0.738	0.744	0.741
MMPNet$^{\dag }$	**0.751**	**0.719**	0.726	**0.723**	0.777	**0.770**	**0.774**

The experimental results on the CMU-MOSEI dataset, presented in Table 3, further validate MMPNet’s effectiveness in multimodal sentiment classification while maintaining interpretability. The larger scale and diversity of CMU-MOSEI provide additional insights into our model’s robustness and generalizability. MMPNet achieves state-of-the-art performance with an accuracy of 73.4%, surpassing the second-best performer MULT (71.8%) by a margin of 1.6%. This improvement is particularly noteworthy given that MMPNet maintains full interpretability through its prototype-based architecture, while MULT operates as a black-box model. In terms of sentiment-specific metrics, MMPNet demonstrates consistent superiority across all evaluation criteria for both positive and negative sentiment detection. For positive sentiment classification, MMPNet achieves the highest precision (73.3%), recall (71.9%), and F1-score (72.6%), showing balanced performance across these metrics. Similarly, for negative sentiment detection, MMPNet outperforms all baselines with a precision of 73.5%, recall of 74.8%, and F1-score of 74.1%. Comparing against other interpretable approaches, MMPNet substantially outperforms both MURO and IMCF. While IMCF achieves a respectable accuracy of 70.8% and MURO reaches 70.3%, MMPNet’s temporal-multimodal prototype learning mechanism enables a performance improvement while maintaining interpretability. This demonstrates that our approach addresses the limitations of existing interpretable methods in capturing complex temporal-multimodal patterns. The single-modality baseline results highlight the challenging nature of the CMU-MOSEI dataset, with the textual modality achieving the highest unimodal accuracy of 70.5%. The visual and acoustic modalities show considerably lower performance (61.3% and 58.9% respectively), emphasizing the importance of effective multimodal information fusion. MMPNet’s superior performance indicates its ability to effectively leverage complementary information across modalities while maintaining interpretable representations through its dual-branch prototype architecture. These results, consistent with our findings on CMU-MOSI, demonstrate MMPNet’s robust performance across different datasets and its ability to effectively combine the benefits of high accuracy and model interpretability in multimodal sentiment analysis (Table 3).

**Table 3:** Performance comparison on the CMU-MOSEI dataset, evaluating both classification effectiveness and model interpretability. Results include accuracy (‘Acc.’), precision (‘Prec.’), recall (‘Rec.’), and F$_{1}$ scores for both positive and negative sentiment classifications. The comparison spans single-modality baselines, traditional multimodal approaches, and interpretable models (marked with $^\dag$). Bold and underlined values denote the best and second-best performances respectively.

Method	Acc.	Positive			Negative		
		Prec.	Rec.	F$_{1}$	Prec.	Rec.	F$_{1}$
Textual	0.705	0.703	0.694	0.699	0.707	0.715	0.711
Visual	0.613	0.599	0.638	0.618	0.629	0.589	0.608
Acoustic	0.589	0.580	0.587	0.583	0.599	0.592	0.595
EF-LSTM	0.688	0.686	0.689	0.687	0.690	0.688	0.689
LF-LSTM	0.701	0.696	0.712	0.704	0.707	0.691	0.699
EF-Transformer	0.706	0.700	0.705	0.703	0.712	0.706	0.709
LF-Transformer	0.715	0.709	0.709	0.709	0.720	0.720	0.720
RAVEN	0.677	0.676	0.678	0.677	0.679	0.677	0.678
MFN	0.686	0.682	0.693	0.687	0.690	0.679	0.685
MULT	0.718	0.717	0.706	0.711	0.718	0.730	0.724
MURO$^{\dag }$	0.703	0.696	0.707	0.701	0.711	0.700	0.705
IMCF$^{\dag }$	0.708	0.703	0.705	0.704	0.712	0.711	0.711
MMPNet$^{\dag }$	**0.734**	**0.733**	**0.719**	**0.726**	**0.735**	**0.748**	**0.741**

Analysis of fusion strategies reveals that late fusion approaches (LF-Transformer and LF-LSTM) generally achieve better performance than their early fusion counterparts (EF-Transformer and EF-LSTM). This pattern suggests the importance of allowing modality-specific feature learning before integration, which aligns with MMPNet’s architectural design of using separate local prototype branches for each modality. An interesting observation emerges regarding the trade-off between interpretability and performance^56^. While previous interpretable models like MURO and IMCF showed reduced performance compared to black-box models like MULT, MMPNet successfully bridges this gap. It achieves superior performance while maintaining interpretability through its prototype-based architecture, challenging the conventional wisdom about accuracy-interpretability trade-offs. The performance difference across datasets yields additional insights. Despite CMU-MOSI being smaller than CMU-MOSEI, MMPNet shows particularly strong results on CMU-MOSI, potentially benefiting from the inherent few-shot learning capabilities of prototype networks^57^. This suggests that MMPNet’s prototype-based approach may be especially valuable for domains with limited training data. These findings collectively validate MMPNet’s novel approach to multimodal sentiment analysis, demonstrating that temporal-multimodal prototype learning can effectively combine high performance with interpretability.

### Comparison on model efficiency

Analysis of model complexity reveals that MMPNet achieves its superior performance while maintaining efficiency across parameters, floating-point operations per second (FLOPs), and time cost compared to other interpretable approaches. Note that the training and inference time metrics are measured based on experiments conducted on the CMU-MOSEI dataset with a batch size of 256.

As shown in Table 4, MMPNet requires only 236,694 trainable parameters, representing a substantial reduction in model complexity compared to existing interpretable solutions. This efficiency extends to computational operations: MMPNet exhibits significantly lower FLOPs (11,196,780) and shorter computational time (3.6 seconds) compared to MURO, which incurs 1,590,860,066 FLOPs and 43.5 seconds of training and inference time, indicating substantially reduced computational overhead during both training and inference phases. The comparison highlights a dramatic difference in model size between MMPNet and MURO. While MURO employs over 30.9 million parameters and incurs high computational cost with 1.59 billion FLOPs, MMPNet achieves better performance with just 236,694 parameters and 11.2 million FLOPs—a reduction of more than 99%. This decrease in parameter count and FLOPs suggests that MMPNet’s dual-branch prototype architecture more efficiently captures temporal-multimodal patterns than MURO’s routing-based approach. When compared to IMCF, which uses 332,609 parameters, 20.2 million FLOPs, and 7 seconds of computational time, MMPNet maintains competitive efficiency with approximately 29% fewer parameters, 44% lower FLOPs, and 49% faster time while delivering superior performance. The reduction of approximately 29% in parameter count demonstrates that MMPNet’s architectural design effectively balances model capacity with computational efficiency. This efficiency likely stems from the prototype learning mechanism, which enables the model to capture meaningful temporal-multimodal patterns without requiring excessive parameters.

The combination of higher accuracy and lower parameter count has important practical implications. MMPNet’s smaller model size suggests reduced computational requirements for both training and inference, making it more suitable for deployment in resource-constrained environments. Additionally, the lower parameter count may contribute to better generalization capabilities, as evidenced by MMPNet’s strong performance across different datasets. These results demonstrate that MMPNet successfully addresses a major challenge in interpretable machine learning: achieving high performance and interpretability while maintaining model efficiency. The reduction in parameters, FLOPs, and time cost without compromising effectiveness validates our architectural design choices and the efficiency of prototype-based learning for multimodal sentiment analysis.

**Table 4:** Efficiency comparison among inherently interpretable multimodal sentiment analysis models.

**Model**	**Parameter Count**	**FLOPs**	**Training and Inference Time (s)**
MURO	30,902,014	1,590,860,066	43.5
IMCF	332,609	20,212,739	7
MMPNet	**236,694**	**11,196,780**	**3.6**

Significant values are in bold.

### Ablation study

To evaluate the contribution of each architectural component to MMPNet’s performance, we conduct comprehensive ablation experiments using several model variants. We systematically remove or isolate different components while maintaining the core prototype-based learning framework to understand their individual and combined effects on sentiment classification accuracy. The first set of variants examines the impact of individual modality-specific local prototype branches. MMPNet-T retains only the textual local prototype branch while removing visual and acoustic components. Similarly, MMPNet-V and MMPNet-A isolate the visual and acoustic local prototype branches respectively. These single-modality variants help quantify the contribution of each modality’s prototype-based features to the overall sentiment analysis task. To investigate the effectiveness of our dual-branch architecture, we create two additional variants that separate the local and global prototype mechanisms. MMPNet-LPN preserves all three local prototype branches (textual, visual, and acoustic) while removing the global prototype network, allowing us to assess how well modality-specific prototypes perform without cross-modal integration. Conversely, MMPNet-GPN retains only the global prototype branch, enabling evaluation of cross-modal prototype learning in isolation from modality-specific features (Table 5).

**Table 5:** Ablation study results on the CMU-MOSI dataset evaluating the contribution of different components and modalities.

Method	Acc.	Positive			Negative		
		Prec.	Rec.	F$_{1}$	Prec.	Rec.	F$_{1}$
MMPNet-T	0.721	0.657	0.650	0.653	0.765	0.770	0.767
MMPNet-V	0.547	0.455	0.639	0.532	0.663	0.482	0.558
MMPNet-A	0.534	0.452	0.690	0.546	0.673	0.433	0.527
MMPNet-LPN	0.730	0.684	**0.739**	0.710	0.774	0.723	0.748
MMPNet-GPN	0.734	0.712	0.684	0.698	0.752	**0.776**	0.764
MMPNet	**0.751**	**0.719**	0.726	**0.723**	**0.777**	0.770	**0.774**

Significant values are in bold.

Ablation study results on the CMU-MOSI dataset, presented in Table 5, reveals insights about the contribution of each component in MMPNet’s architecture. The single-modality variants demonstrate the relative importance of different input modalities. MMPNet-T, which relies solely on textual prototypes, achieves an accuracy of 72.1%, outperforming the visual (54.7%) and acoustic (53.4%) variants. This substantial performance gap indicates that textual features carry the strongest sentiment signals, aligning with previous findings in multimodal sentiment analysis. However, the marked decrease from the full model’s 75.1% accuracy suggests that textual information alone is insufficient for optimal performance. Examining the dual-branch architecture components, both MMPNet-LPN and MMPNet-GPN show strong performance, achieving accuracies of 73.0% and 73.4% respectively. MMPNet-LPN, which utilizes only local prototype branches, demonstrates particularly strong recall for positive sentiment (73.9%), suggesting that modality-specific prototypes effectively capture fine-grained emotional patterns. Meanwhile, MMPNet-GPN, employing only the global prototype branch, exhibits robust performance in negative sentiment detection with the highest recall (77.6%), indicating that cross-modal prototype learning effectively captures more complex sentiment patterns. The full MMPNet architecture, combining both local and global prototype branches, achieves the highest overall accuracy of 75.1% and consistently strong performance across all metrics. This comprehensive performance validates our architectural design choice of incorporating both modality-specific and cross-modal prototype learning. The results demonstrate that the combination of local and global prototype branches creates complementary effects, enabling more robust and accurate sentiment classification than either component in isolation (Table 6).

**Table 6:** Ablation study results on the CMU-MOSEI dataset evaluating the contribution of different components and modalities.

Method	Acc.	Positive			Negative		
		Prec.	Rec.	F$_{1}$	Prec.	Rec.	F$_{1}$
MMPNet-T	0.714	0.710	0.703	0.706	0.717	0.724	0.720
MMPNet-V	0.605	0.593	0.620	0.606	0.618	0.591	0.604
MMPNet-A	0.587	0.575	0.603	0.589	0.600	0.571	0.585
MMPNet-LPN	0.721	0.718	0.709	0.713	0.723	0.732	0.728
MMPNet-GPN	0.726	0.715	**0.734**	0.724	**0.738**	0.718	0.728
MMPNet	**0.734**	**0.733**	0.719	**0.726**	0.735	**0.748**	**0.741**

Significant values are in bold.

The ablation experiments conducted on the larger CMU-MOSEI dataset, detailed in Table 6, provide additional insights into the effectiveness of MMPNet’s components while reinforcing findings from the CMU-MOSI experiments. Among the single-modality variants, MMPNet-T achieves the strongest performance with 71.4% accuracy, demonstrating the primacy of textual information in sentiment analysis. However, both visual and acoustic modalities show meaningful contributions, with MMPNet-V and MMPNet-A achieving accuracies of 60.5% and 58.7% respectively. These results validate the importance of incorporating multiple modalities while highlighting the particular significance of textual features in sentiment classification tasks. Examination of the architectural components reveals strong performance from both specialized branches. MMPNet-LPN, utilizing only local prototype branches, achieves 72.1% accuracy with balanced performance across positive and negative sentiments. MMPNet-GPN shows even stronger results at 72.6% accuracy, with notably high precision for negative sentiment (73.8%) and recall for positive sentiment (73.4%). This suggests that the global prototype branch effectively captures cross-modal sentiment patterns in the larger, more diverse CMU-MOSEI dataset. The complete MMPNet architecture demonstrates superior performance across all primary metrics, achieving 73.4% accuracy and the highest F1-scores for both positive (72.6%) and negative (74.1%) sentiments. This comprehensive improvement over both specialized variants underscores the synergistic relationship between local and global prototype branches. The results again indicate that the combination of modality-specific and cross-modal prototype learning creates a more robust framework for sentiment analysis, particularly beneficial when handling larger-scale datasets with diverse expression patterns. These findings align with and extend the insights gained from the CMU-MOSI experiments, providing strong evidence for the effectiveness of MMPNet’s dual-branch architectural design across different dataset scales and characteristics.

### Interpretability analysis

**Fig. 4:**
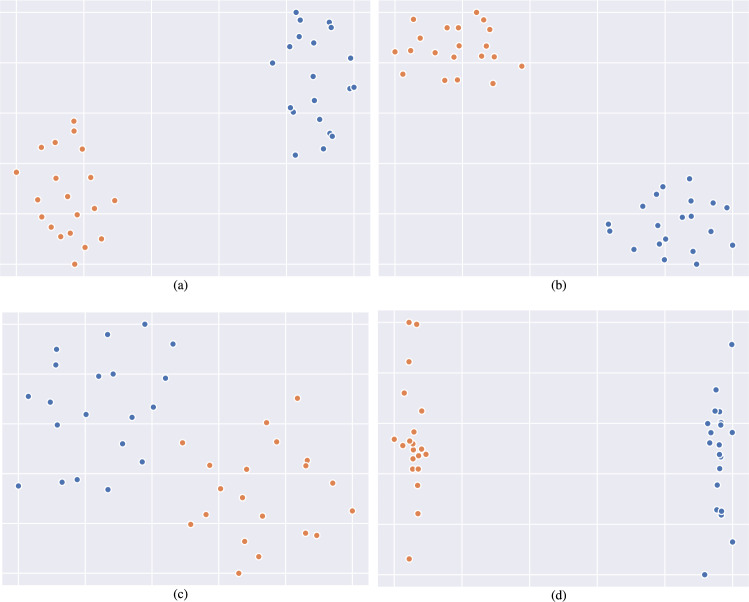
Visualization of learned prototype representations on the CMU-MOSI dataset using t-SNE dimensionality reduction. The plots demonstrate MMPNet’s ability to learn discriminative features, with clear separation between positive (blue) and negative (orange) sentiment prototypes. (**a**) Textual prototypes $\mathscr {P}_{t}$ show distinct clustering patterns, indicating effective learning of sentiment-specific language features. (**b**) Visual prototypes $\mathscr {P}_{v}$ demonstrate well-defined separation, reflecting the model’s capacity to capture meaningful visual sentiment cues. (**c**) Acoustic prototypes $\mathscr {P}_{a}$ display structured organization of speech-related sentiment patterns, though with some overlap reflecting the inherent complexity of acoustic sentiment analysis. (**d**) Global multimodal prototypes $\mathscr {P}_{m}$ exhibit the sharpest separation, demonstrating the effectiveness of MMPNet’s dual-branch architecture in integrating complementary information across modalities. The consistent clustering behavior across all modalities validates the prototype learning mechanism’s ability to capture interpretable sentiment patterns in the CMU-MOSI dataset.

**Fig. 5:**
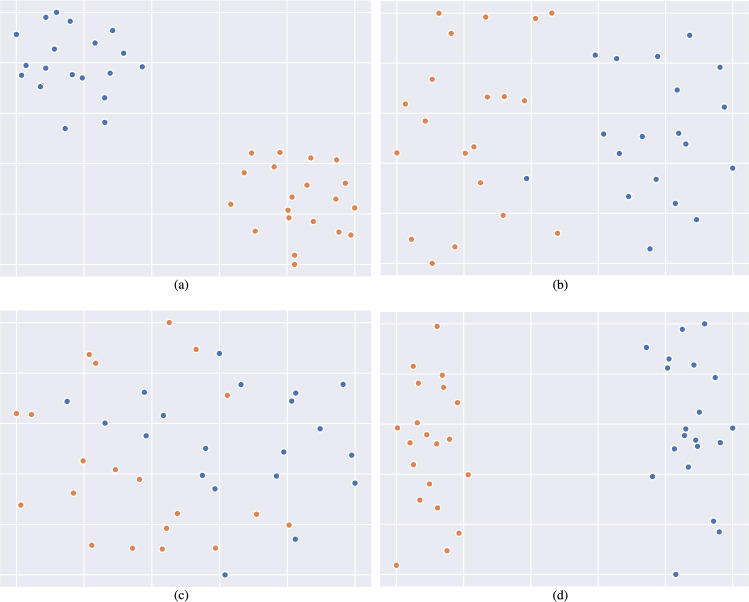
t-SNE visualizations of learned prototype representations on the CMU-MOSEI dataset, demonstrating MMPNet’s ability to learn discriminative features for sentiment classification. Each subplot shows the 2D projection of prototype embeddings, with blue and orange points representing positive and negative sentiment prototypes respectively. (**a**) Textual prototypes $\mathscr {P}_{t}$ exhibit clear clustering by sentiment, indicating effective capture of linguistic patterns. (**b**) Visual prototypes $\mathscr {P}_{v}$ show distinct grouping, reflecting learned representations of image features such as facial expressions and gestures. (**c**) Acoustic prototypes $\mathscr {P}_{a}$ demonstrate separation between sentiment classes based on speech characteristics. (**d**) Multimodal prototypes $\mathscr {P}_{m}$ from the global branch display enhanced separation, suggesting successful integration of cross-modal features. The clear separation between sentiment clusters across all modalities validates MMPNet’s prototype learning mechanism and its effectiveness in capturing modality-specific and integrated sentiment patterns.

To evaluate MMPNet’s interpretability capabilities, we conducted a detailed analysis of the learned prototype representations using dimensionality reduction techniques. Specifically, we visualized the latent prototype feature spaces for both CMU-MOSI and CMU-MOSEI datasets using t-Distributed Stochastic Neighbor Embedding (t-SNE)^58^, which projects high-dimensional representations into a 2D space while preserving relative distances between data points. The visualization results, presented in Figs. 4 and 5, reveal distinct clustering patterns in the prototype feature space. For both datasets, the prototypes naturally segregate into two well-defined clusters corresponding to positive sentiment (shown in blue) and negative sentiment (shown in orange). This clear separation indicates that MMPNet successfully learns discriminative prototype representations that capture fundamental differences between sentiment classes. The emergence of these coherent clusters provides strong evidence for MMPNet’s interpretability. Rather than operating as a black box, the model learns meaningful prototypical patterns that align with human-interpretable sentiment categories. This clustering behavior suggests that MMPNet’s prototype-based architecture effectively identifies and leverages key temporal-multimodal patterns that characterize different emotional expressions in the data. Furthermore, the consistency of these clustering patterns across both datasets demonstrates the robustness of MMPNet’s prototype learning mechanism and its ability to capture generalizable sentiment-related features.

**Fig. 6:**
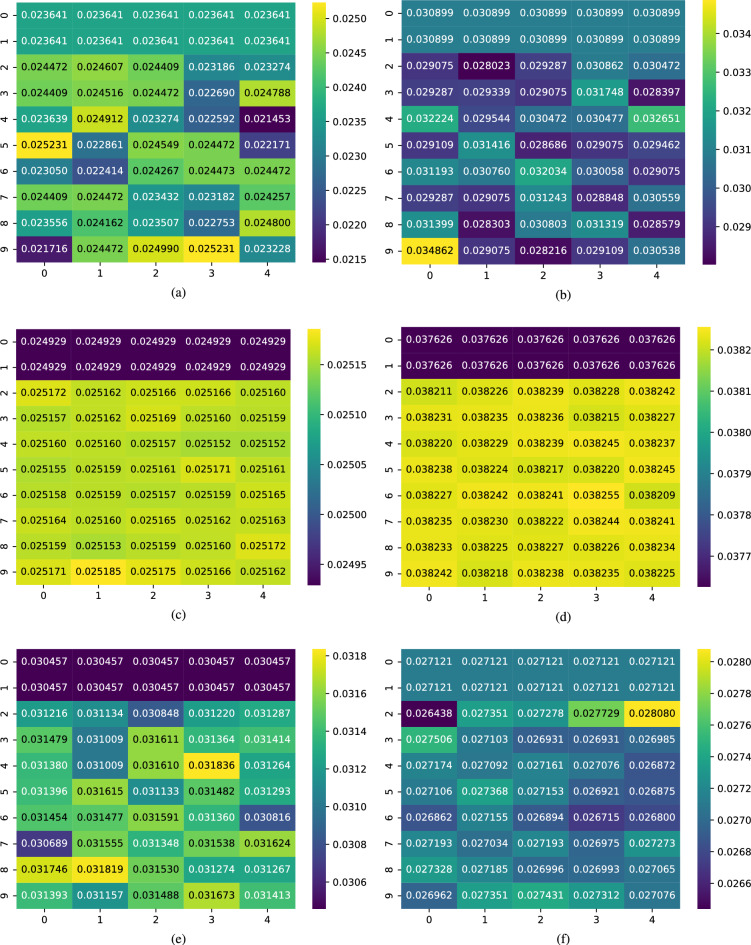
Visualization of similarity matrices showing temporal-modal prototype matching in MMPNet. Each heatmap displays the computed similarity scores between prototypes and encoded sequences for different modality-sentiment combinations. The left column (**a**,**c**,**e**) shows negative sentiment prototypes, while the right column (**b**,**d**,**f**) shows positive sentiment prototypes. Rows correspond to different modalities: textual (**a**,**b**), acoustic (**c**,**d**), and visual (**e**,**f**). Higher values (lighter colors) indicate stronger alignment between temporal segments and learned prototypes. For each modality $m \in \{t,a,v\}$, similarities are computed between prototypes $\textbf{p}_m^i \in \mathscr {P}_m$ and encoded sequences $\mathscr {Z}_m$. The varying similarity patterns across modalities and sentiment classes demonstrate MMPNet’s ability to capture distinct temporal-modal features for sentiment classification.

Another important aspect of MMPNet’s interpretability is its ability to reveal relationships between temporal features and model decisions through similarity analysis. We examine these relationships by analyzing the similarity matrices between learned prototypes $\textbf{p}^i_{m} \in \mathscr {P}{m}$ and encoded sequences $\mathscr {Z}{m}$. The similarity scores are computed using the distance-based formulation from our prototype matching mechanism:

11 $$\begin{aligned} \mathscr {G}_{\textbf{p}^i_m}(\mathscr {Z}_m) = \max _{\textbf{z}^j_m \in \texttt {Patches}(\mathscr {Z}_m)} \cdot \log \Big (\frac{|\textbf{z}^j_m - \textbf{p}^i_m|^2_2 + 1}{|\textbf{z}^j_m - \textbf{p}^i_m|^2_2 + \varepsilon }\Big ). \end{aligned}$$

As visualized in Fig. 6, we present similarity matrices for both negative and positive sentiment prototypes across all three modalities (textual, acoustic, and visual). The left column displays similarity values between a representative negative sentiment prototype and multimodal feature sequences, while the right column shows corresponding similarities for a positive sentiment prototype. Each matrix element represents the similarity between the prototype and a specific temporal segment of the input sequence. The similarity scores quantify how strongly each temporal segment aligns with the prototype patterns learned by the model. Higher similarity values indicate greater influence on the model’s decision-making process, effectively highlighting which parts of the input sequence most strongly activate specific sentiment prototypes. This analysis demonstrates MMPNet’s ability to not only capture multimodal temporal patterns but also provide transparent insights into their relative importance for sentiment classification. By examining these similarity matrices, we can construct ranked lists $\mathscr {R}_{m}$ with respect to modality $m\in \{t,v,a\}$ that order temporal segments according to their contribution to the final prediction, offering fine-grained interpretability of the model’s decision-making process across different modalities and time steps. This temporal-level interpretability distinguishes MMPNet from previous approaches that typically only provide modality-level explanations. The similarity matrices reveal how the MMPNet integrates information across both time and modalities, offering valuable insights into which temporal segments and modalities are most influential for different sentiment classifications.

**Fig. 7:**
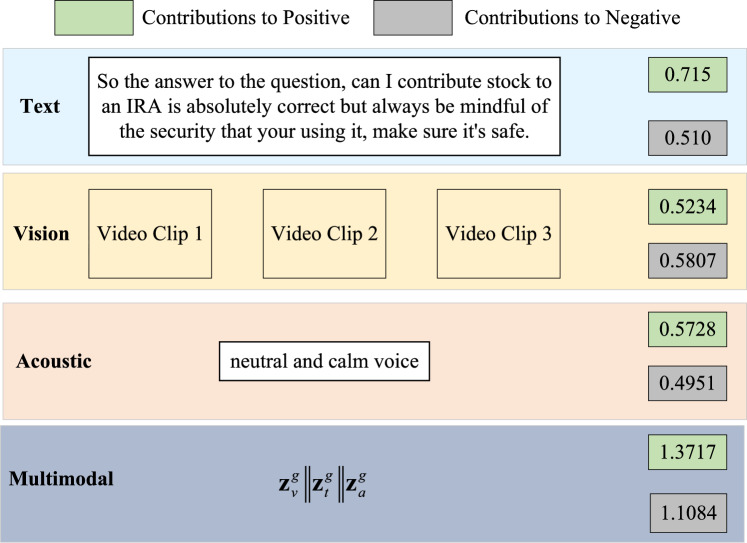
Case study demonstrating MMPNet’s comprehensive modality-level interpretability. The figure shows contribution scores for both positive (green) and negative (gray) sentiment across textual, visual, acoustic, and combined multimodal features. The analysis examines a video clip where the speaker discusses IRA contributions with a neutral tone. The textual modality shows stronger positive sentiment (0.715) compared to negative (0.510), while visual features indicate slightly stronger negative sentiment (0.5807 vs. 0.5234). The acoustic features, characterized by a neutral and calm voice, show balanced contributions. The multimodal integration ($\textbf{z}^g_v|\textbf{z}^g_t|\textbf{z}^g_a$) demonstrates enhanced discriminative power with clearly differentiated positive (1.3717) and negative (1.1084) sentiment scores, highlighting the effectiveness of MMPNet’s prototype-based fusion mechanism.

To generate final predictions, MMPNet extracts the maximum similarity value from each prototype-sequence comparison matrix. For our experiments, we allocated 20 prototypes ($K = 20$) per modality for each sentiment class. Taking the textual modality as an example, the model computes two key arrays: similarity values and their corresponding temporal indices. For negative sentiment, the similarity values range from 0.0207 to 0.0285, with temporal indices spanning positions 11 to 47 in the input sequence. For positive sentiment, the values range from 0.0291 to 0.0464, occurring primarily at temporal positions 18 to 45. This granular analysis reveals how MMPNet achieves both temporal and modality-level interpretability. By aggregating similarity values across prototypes within each modality, we can quantify each modality’s relative contribution to both negative and positive sentiment classifications. To illustrate this capability, we present a detailed case study in Fig. 7 that visualizes these modality-specific contributions. The case study demonstrates how similarity scores serve as confidence indicators for sentiment classification, where higher aggregated values suggest stronger alignment with a particular sentiment category. However, it’s important to note that these values represent relative contributions rather than direct probabilities. This dual-level interpretability, *i*.*e*. at both temporal and modality levels, distinguishes MMPNet from previous approaches that typically focus on just one aspect of model behavior.

### Hyperparameter analysis

**Fig. 8:**
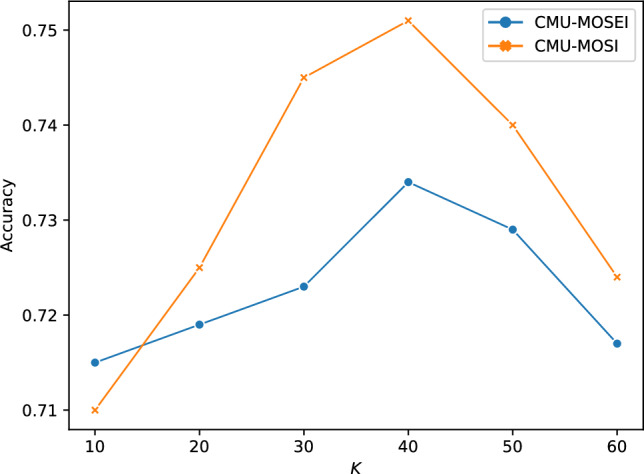
Impact of the number of prototypes (*K*) on classification accuracy for CMU-MOSI and CMU-MOSEI datasets.

The number of prototypes (*K*) represents a an important hyperparameter in MMPNet, as it determines the model’s capacity to capture distinct temporal-multimodal patterns. To evaluate MMPNet’s sensitivity to this parameter, we conducted experiments varying *K* from 10 to 60 in increments of 10, measuring classification accuracy on both CMU-MOSI and CMU-MOSEI datasets. As shown in Fig. 8, the MMPNet’s performance demonstrates a clear relationship with the prototype count. Initially, accuracy improves substantially as *K* increases from 10 to 40, suggesting that a larger prototype set enables the model to capture more nuanced sentiment patterns across modalities. However, performance begins to degrade when *K* exceeds 40, likely due to overfitting as the model attempts to learn too fine-grained distinctions that may not generalize well to unseen data. This empirical analysis guided our selection of $K=40$ as the optimal value, balancing the model’s capacity to capture meaningful temporal-multimodal patterns while maintaining generalization capabilities. The observed sensitivity to *K* underscores the importance of careful hyperparameter tuning in prototype-based multimodal sentiment analysis.

## Conclusions

We address the gap in interpretable multimodal sentiment analysis by introducing MMPNet, a novel framework that extends beyond traditional modality-level interpretability to incorporate temporal dynamics. While existing approaches have focused primarily on understanding modality-specific contributions, our prototype-based architecture enables interpretation of both temporal patterns and cross-modal interactions in sentiment classification decisions. MMPNet builds upon the ProtoPNet framework to deliver dual-level interpretability. Specifically, it identifies important temporal segments within each modality while also quantifying the relative importance of different modalities in the final prediction. Through extensive experimentation on CMU-MOSI and CMU-MOSEI datasets, we demonstrate that MMPNet achieves state-of-the-art performance while maintaining full interpretability and parameter efficiency. The framework’s flexible design allows for extension to additional modalities and adaptation to similar multimodal analysis tasks.

Despite these advances, several limitations inspire future research. First, unlike the original ProtoPNet, MMPNet cannot generate reconstructed visualizations from learned prototypes due to the inherent complexity of temporal-multimodal data. Second, the current architecture is specifically designed for classification tasks, limiting its applicability to regression problems common in affective computing. Future work will focus on addressing these limitations through enhanced visualization methods and architectural modifications to support continuous-valued predictions while maintaining interpretability.

## Data Availability

The datasets supporting the results of this study are available in the CMU-MultimodalSDK repository (i.e., https://github.com/CMU-MultiComp-Lab/CMU-MultimodalSDK)
